# Gene expression profiling of DBA/2J mice cochleae treated with l-methionine and valproic acid

**DOI:** 10.1016/j.gdata.2015.06.022

**Published:** 2015-07-02

**Authors:** Fuyuki Miya, Hideki Mutai, Masato Fujii, Keith A. Boroevich, Tatsuo Matsunaga, Tatsuhiko Tsunoda

**Affiliations:** a Laboratory for Medical Science Mathematics, RIKEN Center for Integrative Medical Sciences, Yokohama, Japan; b Laboratory of Auditory Disorders, Division of Balance and Hearing Research, National Institute of Sensory Organs, National Tokyo Medical Center, Tokyo, Japan; c Division of Balance and Hearing Research, National Institute of Sensory Organs, National Tokyo Medical Center, Tokyo, Japan

**Keywords:** Hearing loss, Microarray, DBA/2J mouse, l-Methionine, Valproic acid

## Abstract

DBA/2J mice, which have homozygous mutations in *Cdh23* and *Fscn2*, are characterized by early onset hearing loss at as early as three-weeks of age (Noben-Trauth et al., 2003 [1]) and are an animal model for progressive hearing loss research. Recently, it has been reported that epigenetic regulatory pathways likely play an important role in hearing loss (Provenzano and Domann, 2007 [2]; Mutai et al., 2009 [3]; Waldhaus et al., 2012 [4]). We previously reported that DBA/2J mice injected subcutaneously with a combination of epigenetic modifying reagents, l-methionine (MET) as methyl donor and valproic acid (VPA) as a pan-histone deacetylases (Hdac) inhibitor, showed a significant attenuation of progressive hearing loss by measuring their auditory brainstem response (ABR) thresholds (Mutai et al., 2015 [5]). Here we present genome wide expression profiling of the DBA/2J mice cochleae, with and without treatment of MET and VPA, to identify the genes involved in the reduction of progressive hearing loss. The raw and normalized data were deposited in NCBI's Gene Expression Omnibus (GEO ID: GSE62173) for ease of reproducibility and reanalysis.

**Table:** 

Specifications	
Organism/cell line/tissue	*Mus musculus* (DBA/2J)
Sex	Male
Sequencer or array type	Affymetrix GeneChip Mouse Genome 430 2.0 Array
Data format	Raw data: CEL files, normalized data: SOFT, MINiML and TXT files
Experimental factors	DBA/2J mice at 4 weeks of age (untreated), 12 week old mice treated with control vehicle (0.1 M sodium bicarbonate) for 8 weeks, and 12 week old mice treated with l-methionine and valproic acid were analyzed.
Experimental features	Microarray gene expression profiling to identify transcripts that are regulated by l-methionine and valproic acid in cochleae of DBA/2J mice as an animal model for hearing loss
Consent	Not applicable
Sample source location	Mice were purchased from Clea Japan (Tokyo, Japan).

## 1 Direct link to deposited data

The deposited data can be found at: http://www.ncbi.nlm.nih.gov/geo/query/acc.cgi?acc=GSE62173.

## 2 Experimental design, materials and methods

DBA/2J mice, which have homozygous mutations in Cdh23 and Fscn2, are characterized by early onset hearing loss as early as three-weeks of age [1] and are an animal model for progressive hearing loss research. Recently, it has been reported that epigenetic regulatory pathways likely play an important role in hearing loss [2], [3], [4]. We previously reported that DBA/2J mice injected subcutaneously with a combination of epigenetic modifying reagents, l-methionine (MET) as a methyl donor and valproic acid (VPA) as a pan-histone deacetylase (Hdac) inhibitor, showed a significant attenuation of progressive hearing loss by measuring their auditory brainstem response (ABR) thresholds [5]. Here we investigated genome wide expression profiling of the DBA/2J mice cochleae, with and without treatment of MET and VPA, to identify the genes involved in the reduction of progressive hearing loss.

### 2.1 Animals and treatment

**Fig. 1:**
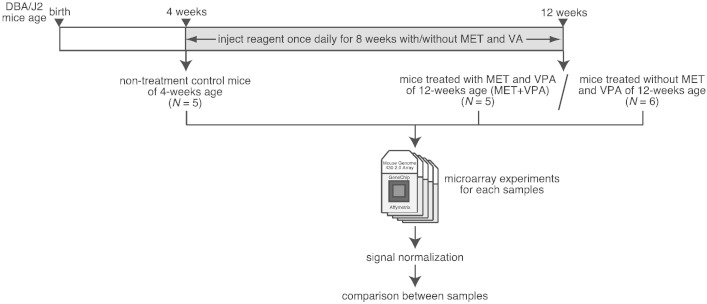
Workflow of the experiments and analyses.

DBA/2J mice were purchased from Clea Japan (Tokyo, Japan). Mice were housed in plastic cages with metallic lid in a room with a 12-h light/dark cycle and 55% humidity at 23 °C and had free access to food and water. Male DBA/2J mice at 4-weeks postnatal age were used as non-treatment young controls (Fig. 1). In vitro treatment mice with epigenetic modifying reagents were male DBA/2J mice of 4-weeks of age subcutaneously injected once daily for 8 weeks with l-methionine (MET; 500 mg/kg/day, Wako Pure Chemicals, Osaka, Japan) and valproic acid (VPA; 300 mg/kg/day, Sigma-Aldrich, MO, USA) in 0.1 M sodium bicarbonate (10 ml/kg body weight) (MET + VPA, Fig. 1). The age-matched control mice were male DBA/2J mice of 4-weeks old subcutaneously injected once daily for 8 weeks with only vehicle (0.1 M sodium bicarbonate) (Fig. 1). The hearing loss phenotype was investigated by measurement of auditory brainstem response (ABR) as previously reported [5]. A significant attenuation effect of progressive hearing loss was observed and validated in mice injected with MET and VPA as previously reported [5]. All experimental procedures were approved by the Institutional Animal Care and Use Committee of National Tokyo Medical Center (permit number: 12-animal-02). The experiments were carried out in accordance with the approved guidelines.

### 2.2 Microarray experiments

Total RNA was extracted from the whole left cochleae of mice using TRIzol reagent (Life Technologies, CA, USA) and further purified using the RNeasy Mini Kit (Qiagen, Hilden, Germany) according to the manufacturer's instruction. The quality of the RNA was assessed using Agilent 2100 Bioanalyzer (Agilent Technologies, CA, USA). We used only the high quality RNA (RIN score > 7), derived from 5 to 6 mice in each group, for microarray experiments (Fig. 1). For microarray hybridization, the biotinylated aRNA was prepared according to the standard Affymetrix protocol using GeneChip 3′ IVT Express Kit (Affymetrix, CA, USA) from the 250 ng total RNA. Then, 10 μg of aRNA was hybridized for 16 h at 45 °C on GeneChip Mouse Genome 430 2.0 Array (Affymetrix) with 45,101 probes. The GeneChips were washed and stained in the Affymetrix Fluidics Station 450. The stained GeneChips were scanned using the Affymetrix Scanner 3000 7G, the images were digitalized using GeneChip Operating Software (GCOS) v.1.3 (Affymetrix), and the data were exported as CEL files.

### 2.3 Data normalization

Gene expression array data were normalized using the MAS5 algorithm (Affymetrix). The intensities were converted to a logarithmic scale (base 2). To correct for bias between chips, we then performed quantile normalization [6] to all array data using R software (“affy” and “limma” packages). The signal reliability of each probe was determined based on the MAS5 Call algorithm (Affymetrix), and each probe was assigned to one of three flags (P: present, M: marginal, and A: absent).

### 2.4 Validation of microarray data by qRT-PCR

Among the genes that were highly differentially expressed between the MET + VPA and vehicle control group in microarray data, we selected 8 genes (*Otx1*, *Slc39a4* (also known as *Zip4*), *Calca*, *Pold3*, *Hspa1b*, *Serpinh1*, *Gabra1* and *Gabra2*) to validate the expression levels by qRT-PCR [5]. For the qRT-PCR, the total RNA was reverse transcribed using SuperScript III (Life Technologies, CA, USA) with random hexamer d(N)6 primers. The details were described in our previous report [5].

### 2.5 Differentially expressed genes and cluster analysis

**Fig. 2:**
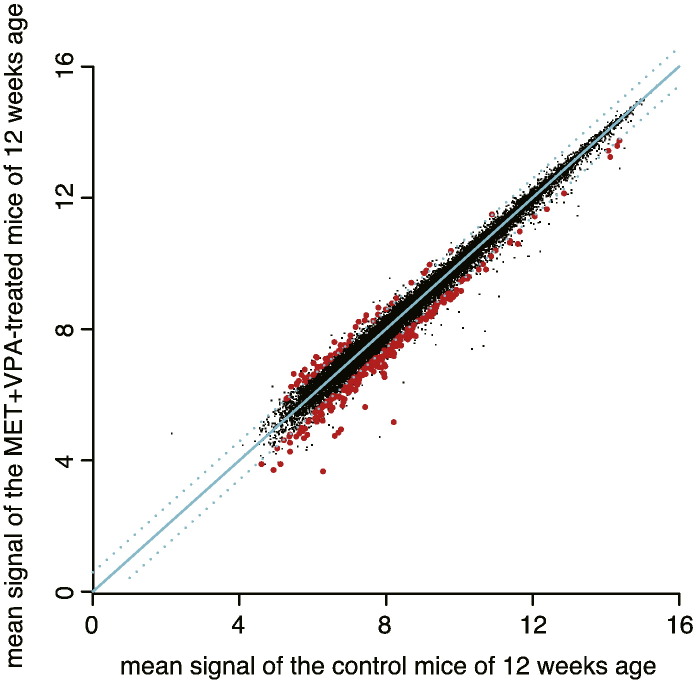
Scatter plot of the microarray data. Mean expression signals of the vehicle-treated control mice of 12 weeks of age against mean expression signals of the MET + VPA-treated mice of 12 weeks of age. The signal values were transformed to a logarithmic scale (base 2). The cyan line shows y = x (no difference), the cyan dotted-lines show a 1.5-hold change. The black and red dots represent non-differentially and differentially expressed probes (*p*-value < 0.05 and fold change ≥ 1.5), respectively.

To identify possible genes involved in attenuation of progressive hearing loss induced by MET and VPA, we compared the cochleae expression of MET + VPA to the vehicle control group using Welch's two-side *t*-test. After selecting only those probes assigned a present (P) flag for all samples in at least one group for the statistical tests, 24,787 probes remained. Genes were considered differentially expressed with a *p*-value < 0.05 and a fold change ≥ 1.5.Using these criteria, 55 probes (49 genes) and 244 probes (195 genes) were up- and down-regulated in the MET + VPA group, respectively (Fig. 2).

**Fig. 3:**
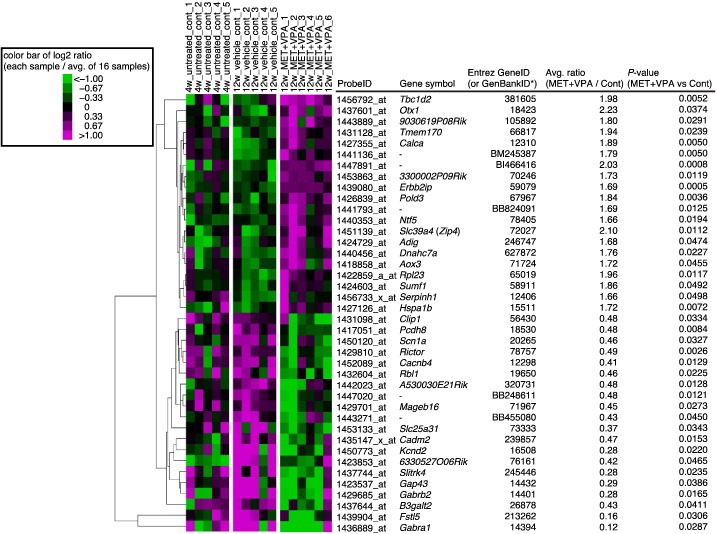
Cluster and statistical analysis results for the top 20 differentially up- or down-regulated genes in the MET + VPA-treated cochleae relative to vehicle-treated control cochleae of 12-week old mice. The cluster analysis was performed using 16 samples (4-weeks non-treated control mice, *N* = 5; 12-weeks vehicle-treated mice, *N* = 5; 12-weeks MET + VPA-treated mice, *N* = 6). *GenBank IDs start with “B”.

Cluster analysis was performed using the average linkage method with Euclidian distance in Cluster and TreeView [7] (Fig. 3).

## 3 Discussion

We present a dataset of microarray expression profiling of the DBA/2J mice cochleae treated with and without an epigenetic modifying reagent to investigate the genes involved in the reduction of progressive hearing loss. In our previous report [5], we particularly focused on *Slc39a4* (also known as zinc importer *Zip4*) from the microarray data. In addition to *Scl39a4*, other genes differentially expressed in the presence of the MET and VPA may contribute to the observed attenuation effect on progressive hearing loss. We believe that this expression profiling dataset would be valuable for further investigating and understanding of the molecular mechanism in hearing loss.

## Conflict of interest

The authors declare no conflict of interests.
